# Neurosyphilis: insights into its pathogenesis, susceptibility, diagnosis, treatment, and prevention

**DOI:** 10.3389/fneur.2023.1340321

**Published:** 2024-01-11

**Authors:** Sirui Wu, Fei Ye, Yuanfang Wang, Dongdong Li

**Affiliations:** Department of Laboratory Medicine, West China Hospital of Sichuan University, Chengdu, China

**Keywords:** neurosyphilis, *Treponema pallidum*, HIV, susceptibility, pathogenesis, antibiotic treatment, prevention

## Abstract

**Background and aim:**

Invasion of the central nervous system by *Treponema pallidum* can occur at any stage of syphilis. In the event that *T. pallidum* is not cleared promptly, certain individuals may experience progression to neurosyphilis, which manifests as cognitive and behavioral abnormalities, limb paralysis, and potentially fatal outcomes. Early identification or prevention of neurosyphilis is therefore crucial. The aim of this paper is to conduct a critical and narrative review of the latest information focusing exclusively to the pathogenesis and clinical management of neurosyphilis.

**Methodology:**

To compile this review, we have conducted electronic literature searches from the PubMed database relating to neurosyphilis. Priority was given to studies published from the past 10 years (from 2013 to 2023) and other studies if they were of significant importance (from 1985 to 2012), including whole genome sequencing results, cell structure of *T. pallidum*, history of genotyping, and other related topics. These studies are classic or reflect a developmental process.

**Results:**

Neurosyphilis has garnered global attention, yet susceptibility to and the pathogenesis of this condition remain under investigation. Cerebrospinal fluid examination plays an important role in the diagnosis of neurosyphilis, but lacks the gold standard. Intravenous aqueous crystalline penicillin G continues to be the recommended therapeutic approach for neurosyphilis. Considering its sustained prominence, it is imperative to develop novel public health tactics in order to manage the resurgence of neurosyphilis.

**Conclusion:**

This review gives an updated narrative description of neurosyphilis with special emphasis on its pathogenesis, susceptibility, diagnosis, treatment, and prevention.

## Introduction

Neurosyphilis (NS) is a neurological infection caused by the spirochete *Treponema pallidum*. *T. pallidum* can affect the central nervous system (CNS) during any stage of syphilis (1). Estimates from the WHO indicate that approximately 22.3 million individuals worldwide had *T. pallidum* infection, with 7.1 million new cases, in 2020 (2). However, most regions have not monitored neurosyphilis at the national level, and there are few studies reporting the incidence of neurosyphilis (3). As 1.2% to 1.8% of patients with early syphilis will develop neurosyphilis, there is persistent prevalence of neurosyphilis worldwide (4). In Europe, the estimated annual incidence of neurosyphilis varies from 0.16 to 2.1 per 100 000 adults; in Australia, the annual incidence of neurosyphilis from 2007 to 2016 was 2.47 cases per 10,0000 people; in the Canadian province of British Columbia, the incidence of neurosyphilis increased from 0.03 cases per 10,0000 people in 1992 to 0.8 cases per 10,0000 people in 2012; in Guangdong province of China, the incidence of neurosyphilis increased from 0.21 cases per 10,0000 people in 2009 to 0.31 cases per 10,0000 people in 2014 (5–8). In high-income countries, the prevalence of syphilis is extremely high among men; in low-income and middle-income countries, syphilis is prevalent among the general population; WHO estimates that the prevalence of syphilis in Africa was the highest in 2016, which may be related to insufficient health education, human and infrastructural resources (9–11). It is speculated that similar patterns also exist in patients with neurosyphilis (10). Neurosyphilis not only imposes a great economic burden on patients but also causes a decline in quality of life due to the stigma associated with the disease (12).

As pathogenic treponemes, yaws and syphilis treponemes exhibit genetic similarity of more than 99.8% in their genome (13). However, yaws are unlikely to affect the CNS, suggesting that certain small genetic changes might be responsible for the differences in pathogenesis among these organisms (13). Exploring the genomes, neuroinvasion properties, and transmission routes of *T. pallidum* is essential for comprehending the pathogenesis of neurosyphilis and developing effective clinical strategies. Benzathine penicillin G, the preferred drug for treating primary syphilis, secondary syphilis, and tertiary syphilis patients with normal cerebrospinal fluid (CSF) examination, cannot reach an effective concentration in the CSF (14). Thus, identifying syphilis patients at a high risk of developing neurosyphilis would allow for targeted intervention. While the exact pathogenesis is not completely understood, certain factors, such as genetic susceptibility, for neurosyphilis have been identified (15). Despite this, numerous clinical and preclinical questions remain unanswered due to the complexities of real-world practice. This review describes the strain factors and mechanisms behind the neuroinvasion property of *T. pallidum*, as well as risk factors, predictors, diagnosis, treatment and prevention of neurosyphilis. Additionally, it addresses existing controversies and provides a discussion on future prospects.

To compile this review, we have conducted electronic literature searches from the PubMed database relating to neurosyphilis. Priority was given to studies published from the past 10 years (from 2013 to 2023) and other studies if they were of significant importance (from 1985 to 2012), including whole genome sequencing results, cell structure of *T. pallidum*, history of genotyping, and other related topics. These studies are classic or reflect a developmental process.

## The biological basis of *T. pallidum*

**Figure 1:**
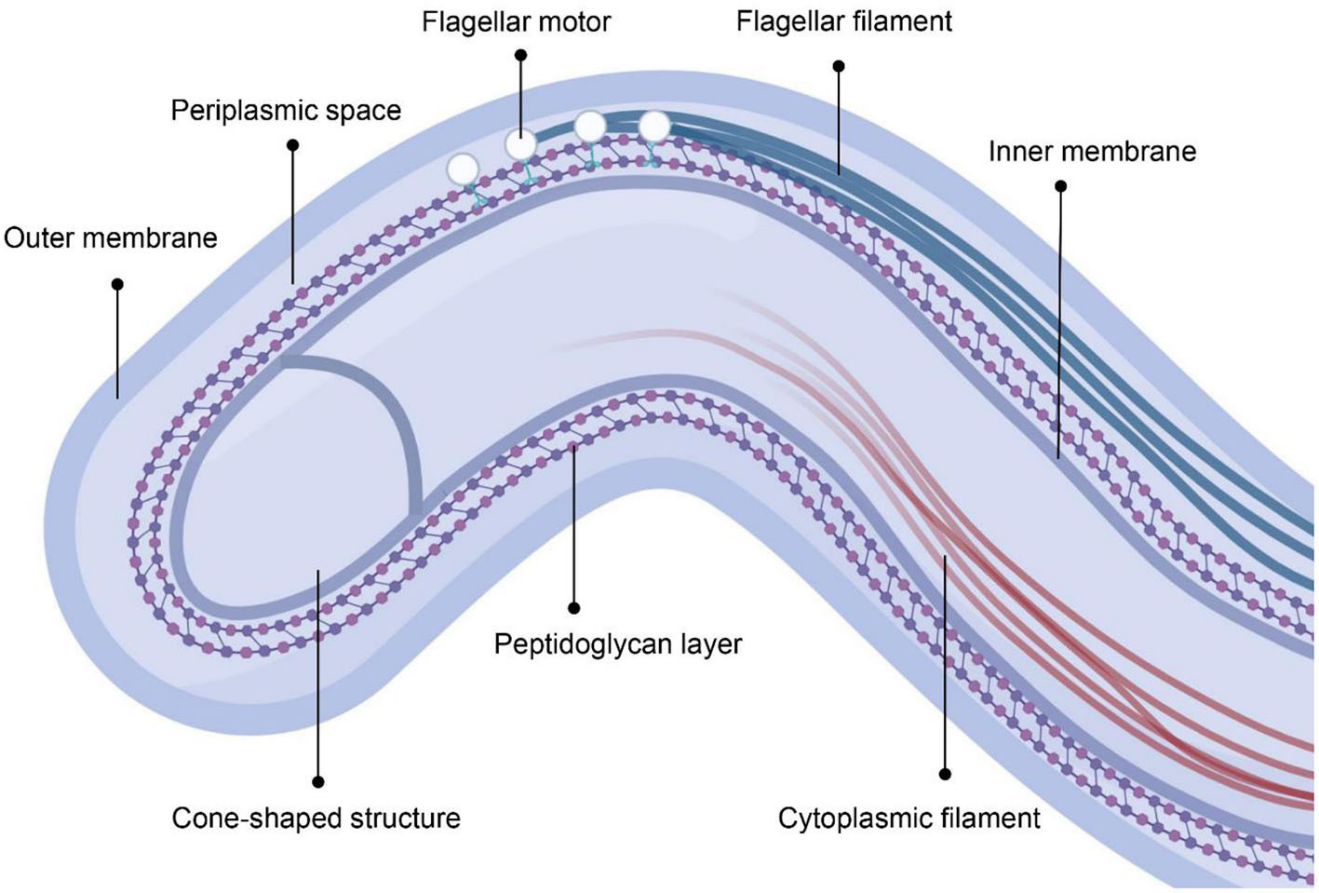
The cellular architecture of *T. pallidum*. The figure was created in BioRender.com.

In contrast to gram-negative bacteria, *T. pallidum* has a helical shape with a fragile dual-membrane structure and peptidoglycan layer, but lacks lipopolysaccharides (LPS) (Figure 1) (16). The flagella motors are fixed in the cytoplasmic membrane and arranged in a row, allowing the flagella filaments to extend into the periplasmic space between the two membranes (17). *T. pallidum* can swim by rolling or undulation of the cell body driven by the rotation of periplasmic flagella and it is the existence of periplasmic flagella maintains its helical shape (18). The cytoplasmic filaments anchored to the inner surface of the cytoplasmic membrane are arranged in a ribbon configuration (17). The cone-shaped structure locates at the end of spirochetes outside of the peptidoglycan layer (17). However, the exact role of cytoplasmic filaments and cone-shaped structure remains unknown (17). The defining characteristic of *T. pallidum* is the low density of membrane-spanning proteins and sufficient lipoproteins, which may reflect its capacity for host immune response evasion (19).

## The mechanisms of invasion of the CNS by *T. pallidum*

Current research suggests that *T. pallidum* does not release toxins into the host, but is one of the most invasive spirochetes (20). It has been demonstrated in rabbit models, mouse models, and human subjects that *T. pallidum* is capable of invading the CNS early in the disease (21). Pham et al. found *T. pallidum* in brain tissue, which was confirmed by 16S ribosomal RNA sequencing (22). Additionally, metagenomic next-generation sequencing (mNGS) detected *T. pallidum* nucleic acids in the CSF at low levels (23). These studies provide substantive evidence for the neuroinvasive capacity of *T. pallidum*.

The presence of *T. pallidum* in the CNS suggests its ability to evade the immune system and actively invade the CNS, leading to sustained damage. Macrophages are critical immune cells, especially microglia in the CNS, acting as the first line of immune defense (24). A case report of neurosyphilis observed severe proliferation of microglia in the cerebral cortex (25). CSF sTREM2 (a biomarker of microglial activation) was observed to be at significantly higher levels in neurosyphilis patients (26). Further study revealed that *T. pallidum* promotes microglial apoptosis and inhibits microglial migration as a means of evading clearance (27, 28). The balance between phagocytic uptake and *T. pallidum* evasion is influenced by production of opsonic antibodies, with TP0326 (BamA) and TP0751 (pallilysin) being identified as opsonic targets (29, 30). In addition, Tp47 can activate the NLPR2 inflammasomes in macrophages through PKM3 dependent glycolysis, thereby mediating the infection of *T. pallidum* (31).

**Figure 2:**
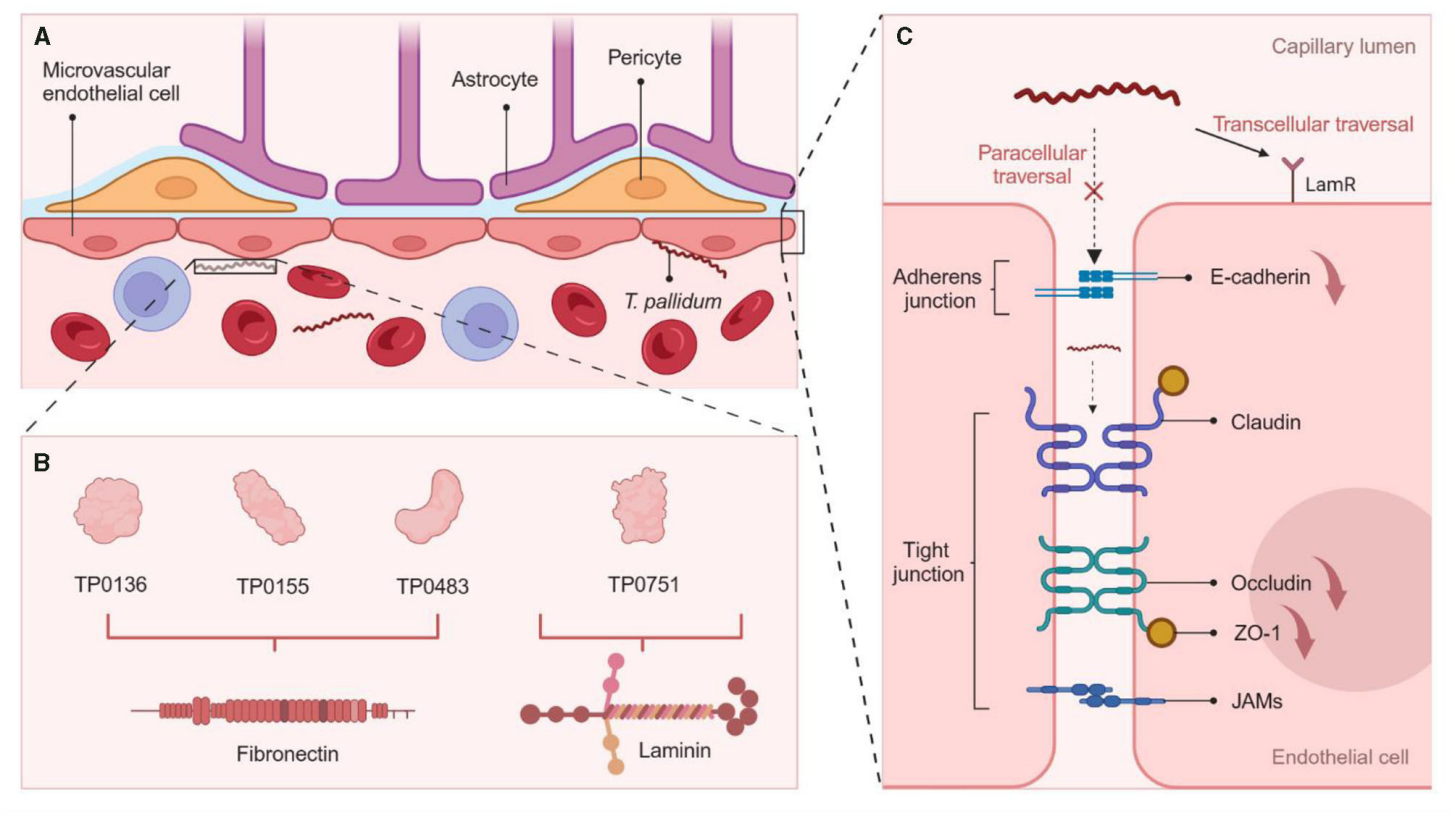
Mechanisms of *T. pallidum* for Crossing the BBB. (**A)** The BBB is composed of brain microvascular endothelial cells, astrocytes, and pericytes. Attaching to the host cells is the first step in *T. pallidum* neuroinvasion (33). (**B)** Several *T. pallidum* proteins are identified to be adhesins, including laminin- (TP0751) and fibronectin- (TP0136, TP0155, TP0483) binding proteins (34). (**C)** *T. pallidum* adheres to endothelial cells at the site of intercellular junction, resulting in a notable reduction in the expression of tight junction proteins ZO-1 and occludin through TP0751, and disrupting VE-cadherin, thereby entering the BBB through a paracellular pathway; TP0751 coordinates intercellular transport by engaging with host receptors (LamR) in lipid rafts and inducing endothelial uptake and transport in a cholesterol-dependent manner (35). The figure was created in BioRender.

*T. pallidum* exhibits various effects on host vascular endothelial cells, potentially linked to its invasion of the CNS (32) (shown in Figure 2). Scanning electron microscopy revealed that *T. pallidum* can directly adhere to human brain microvascular endothelial cells *in vitro* (36). TP0751 shares the same endothelial receptor with other neurotropic pathogens, and thus mediates interactions with endothelial cells (37); TP0751 alters expression of tight junction proteins, influencing the permeability of the blood-brain barrier (BBB) by promoting bEnd3 cell apoptosis and IL-6 secretion (38). Similarly, recombinant TP0965 shows a higher level of endothelial permeability induction (39). Both intercellular junction pathways and lipid raft-mediated endocytosis mechanisms for traversing endothelial barriers were observed in *T. pallidum*, but without disruption of barrier permeability (35). The latter view seems more reliable due to the normal or only slightly increased quotient (Q_alb_) level in neurosyphilis patients in clinical practice (40). In addition to interacting with macrophages and endothelial cells, the biochemical system, antigenic variation system, and matrix metalloproteinases (MMPs)/tissue inhibitors of metalloproteinases (TIMPs) imbalance are involved in the pathogenesis of *T. pallidum* (41–43).

## Association of *T. pallidum* genome, genotypes and neurosyphilis

The *T. pallidum* genome comprises a circular chromosome with a total of 1041 predicted open reading frames (ORFs) (16). Two major *T. pallidum* lineages (Nichols and SS14) cocirculated across multiple continents, with a worldwide predominance of SS14 lineages, which may be explained by its resistance to macrolides (44). Whether this lineage is related to neurosyphilis has not been studied, but the genotype has and is discussed below. Notably, the genome exhibits a distinct set of repeat gene family sequences, particularly the *tprK* gene, which is involved in immune evasion and has potential as a latent vaccine (41). Furthermore, the genome displays a considerable number of motility-associated genes (e.g., *FlaB1, FlaB2, FlaB3, FlaA, FliG*), enabling efficient movement of *T. pallidum* in highly viscous environments such as connective tissues by traveling planar waves (16, 17). The periplasmic flagella and flexible peptidoglycan layer are the structural basis for achieving this movement (17).

Findings from the rabbit intravenous infection model have shown that certain strains of *T. pallidum*, such as Sea 81–4, exhibit a particular affinity for the CNS (45). This finding provides valuable clues regarding the association of strain typing and the neurosyphilis phenotype, and the potential microbiological mechanisms involved. The typing schemes frequently used in *T. pallidum* are CDC typing (CDCT), enhanced CDC typing (ECDCT), and multilocus sequence typing (MLST), based on analysis of restriction fragment length polymorphism (RFLP) or direct sequencing (46, 47). The loci detected in CDCT schemes include the *acidic repeat protein* (*arp*) gene and the *T. pallidum* repeat family genes [*tprE* (*tp0313*), *tprG* (*tp0317*), *tprJ* (*tp0621*)] (48). Compared to CDCT, ECDCT adds the sequence analysis of the *tp0548* gene and has stronger discrimination against strains, while MLST focuses on the analysis of four genes (*tp0136, tp0548, tp0705* and 23S rDNA genes) and further improves the typing resolution of SS14-like strains (48, 49). For ECDCT, consistent observations indicate a higher likelihood of neurosyphilis with strain type 14d/f (Table 1) (47, 48, 50, 51). Correspondingly, MLST type 1.1.2 appears to exhibit greater neuroinvasion (47). Except for South Africa, the majority of the CSF specimens (54%) inspected involved the 14a strain type, though subtype 14d was commonly found in genital ulcer specimens from syphilis patients during the same period (46). However, the nonuniform inclusion criteria of neurosyphilis patients and the lack of enough subjects limit the universality of these studies. Considering that strain type 14d/f is predominant among syphilis patients, the base effect linked to this subtype predisposing toward the development of neurosyphilis should be taken into account (52). A systematic review and meta-analysis concluded that CSF from patients with late neurosyphilis had low typing efficiency (46.4%) (53). The typing efficiency of lesion exudate was higher. However, it was difficult to collect from neurosyphilis patients (47). A more sensitive CSF typing method for *T. pallidum* deserves further investigation. Chen et al. developed a suite of PCR-LwCas13a syphilis assays with excellent sensitivity and specificity, which may be a promising alternative to genotyping (54).

**Table 1:** Overview of six studies on molecular typing of *T. pallidum* strains from neurosyphilis patients.

**First author (citation)**	**Location**	**Specimen collection period**	**Specimen type**	**Specimen size**	**Typing system**	**Strain**
Sahi et al. (47)	Seattle, USA, America	1999–2017	Whole blood, CSF	18	MLST	Eight patients had a 1.1.2 strain; no patients had a 1.3.1 strain; nine patients had a tp0705 type 2 strain.
Sahi et al. (47)	Seattle, USA, America	1999–2017	Whole blood, CSF	18	ECDCT	One patient had a 14d/g strain; 16 patients had a tp0548 type f strain.
Marra et al. (48)	Seattle, USA, America	1999–2008	CSF	84	ECDCT	22 patients had a 14d/f strain.
Dai et al. (50)	Shanghai, China, Asia	2007–2011	Lesion swab	4	ECDCT	Two patients had a 14d/f strain; two patients had a 19d/c strain.
Read et al. (51)	Sydney, Australia, Oceania	2004–2011	Lesion swab	2	ECDCT	Two patients had a 14d/f strain; two of six patients with strain type 14d/f developed neurosyphilis, compared with 0/85 with non-14d/f strains.
Molepo et al. (46)	Pretoria, South Africa, Africa	1999–2000	CSF	50	CDC	Seven patients had a 14a strain; four patients had a 3e strain; one patient had a 17e strain; one patient had a 2i strain.

CSF, cerebrospinal fluid; ECDCT, enhanced CDC-typing; MLST, multi-locus sequence typing.

## Risk factors for neurosyphilis in HIV-negative patients

### Sex and age

An increased prevalence of syphilis has been observed among men in China, the United States, and European countries, with a significant proportion of men having sex with men (MSM) (5, 55–57). For instance, in the United States, from 2008 to 2018, the estimated number of male patients with syphilis rose from 40,300 to 121,000 (55, 58). However, the prevalence of syphilis is not the highest in upper-middle-income and high-income countries (59). On the one hand, wealth can increase the opportunities for MSM to achieve syphilis by increasing regional mobility (59). On the other hand, the convenience of syphilis detection and treatment is convenient, which can reduce the spread of syphilis (59). The prevalence of syphilis depends on the balance of various situations. However, studies conducted in sub-Saharan Africa have shown that women have a higher prevalence of syphilis, which is related to the cultural, economic, and social marginalization of women in the region (60). Male sex has been identified as a correlating risk factor for neurosyphilis, in addition to its epidemiological significance (3, 61, 62). As we strive to better understand the pathophysiology of neurosyphilis, studies investigating the impact of sex hormones on disease development and progression may yield valuable insights (61).

Older age (≥45/60 years) is an independent risk factor for HIV-negative neurosyphilis patients (3, 62). The link involved may be attributable to the longer courses of disease in elderly individuals. Accordingly, aging is accompanied by progressive immunosenescence, which raises the possibility of infections (63). Interestingly, neurosyphilis has been found to correlated with certain neurodegenerative disorders, such as Alzheimer's disease (AD) (64). Pathological evidence has demonstrated that curly fibers discovered in AD correspond to individual spirochetes, and their aggregation in colonies produces similar senile plaques (65).

###  Serum non-treponemal test titer

Previous studies have indicated that non-treponemal tests, such as the rapid plasma reagin (RPR) test, and toluidine red unheated serum test (TRUST), have stronger specificity than treponemal tests in the diagnosis of neurosyphilis (66). This may be explained by the fact that blood-derived anti-treponemal IgG antibodies can cross through the BBB and enter the CSF, thus leading to false-positive results (67). A multivariate analysis revealed that the risk of developing neurosyphilis, but not secondary syphilis, increases with an elevation in serum RPR titer (3). Jiang et al. retrospectively found that HIV-negative syphilis patients with serum TRUST titers ≥1:16 were eight times more susceptible to developing neurosyphilis (68). Of note, patients with a fourfold decrease in serum RPR titers after treatment are more likely to develop asymptomatic neurosyphilis (ANS), indicating a correlation between ANS and treatment failure (69).

### Specific genes carried by the host

Single-nucleotide polymorphisms (SNPs) of immune regulatory genes may influence susceptibility to neurosyphilis. The −1082 GG and −592 CC genotypes of the IL-10 promoter and the TLR1_1805GG, TLR2_2258GA, and TLR6_745CT/TT genotypes are associated with an increased risk of neurosyphilis (15, 70). At present, the majority of risk factors found in studies are immutable, posing challenges in intervening in risk factors to reduce disease risk. It is imperative to further explore factors such as psychosocial and physical activity factors to broaden our understanding of potential prevention.

## Risk factors for neurosyphilis in HIV-positive patients

According to the European guideline on the management of syphilis, there is no increased risk of neurological involvement in HIV-infected patients with early syphilis (57). However, a study that did not distinguish syphilis stages has shown that syphilis patients with HIV infection are more likely to develop neurosyphilis (71). One possible explanation for this is that HIV-positive patients may be more inclined to undergo comprehensive and timely examinations. From a pathophysiological perspective, the CNS is a more immunologically privileged site due to the presence of the BBB and limited movement of immune cells, combined with the decrease in CD4 T cells and meningeal lesions in HIV-positive patients, which weakens the CNS's ability to defend against *T. pallidum* (72, 73).

In addition to male sex, advanced age, and high serological titers, HIV-positive individuals have several other risk factors regarding immune suppression. Numerous studies have reported an increased risk of neurosyphilis in patients with higher viral load and lower CD4(+) T cells count (< 350 cells/μl) compared to the general population (74, 75). *T. pallidum* itself can induce programmed cell death of CD4(+) and CD8(+) T cells with an increase in HIV-ribonucleic acid (RNA) viral load (76, 77). Actually, the synergism of HIV and *T. pallidum* is complicated and lacks sufficient research. It is well acknowledged that the characteristics of neurosyphilis can be confused with HIV infection, as primarily characterized by an increased karyocyte count and protein concentration (78). Antiretroviral therapy (ART) may ameliorate this status, but it is still unclear whether ART-naive patients with syphilis should undergo lumbar puncture (73). However, what is certain is that the absence of ART or syphilis treatment represents a significant risk factor for neurosyphilis (70, 74, 75).

## Predictive indicators for neurosyphilis risks and treatment responses

Predictors can be roughly divided into two types: temporally advanced indicators and heterotopic predictive indicators. Current research on predicting the risk of neurosyphilis primarily focuses on the latter, particularly certain peripheral blood indicators. Li et al. constructed a prediction model incorporating age, serum TRUST titer, and various blood routine indicators, which offers a valuable reference for the empirical treatment of ANS (79). Serum neurofilament light chain (NfL) and the neutrophil-to-lymphocyte ratio (NLR), which serve as markers of neuroaxonal injury and inflammation, respectively, show promise as novel predictors for neurosyphilis (80, 81). Although diagnosis of neurosyphilis necessitates lumbar puncture, peripheral blood predictors can to some extent indicate changes in the CNS, thereby reducing the need for unnecessary invasive procedures (82). Additionally, applications of metabolomics, transcriptomics, mRNA modification, SNPs, and neuroimaging analysis in neurosyphilis have facilitated screening of additional biomarkers (15, 70, 83–86).

To accurately predict the effectiveness of treatment, temporally advanced indicators and heterotopic predictive indicators should both be explored. The serological response, namely, normalization or decrease of the serum RPR titer, can to some degree predict CSF normalization, and the rate of follow-up lumbar puncture may be reduced if neurological symptoms resolve (87, 88). As a fourfold decline in CSF RPR titer is a reliable predictor for treatment efficacy in CSF RPR-positive general paresis patients within 12 months after completing therapy, it is unnecessary to repeat CSF tests for HIV-negative people (14, 89). A recently developed minimal proteomic array not only allows for disease staging, but also monitors response to appropriate treatment, helping to confirm pharmacological cure (90). Furthermore, it is necessary to discuss factors like socioeconomic status, access to healthcare, and cultural influences which may contribute to the outcomes of neurosyphilis in the future. We should mention two conditions related to syphilis treatment: Jarisch-Herxheimer reaction (JHR) and serofast status. JHR is a transient inflammatory phenomenon observed in syphilis patients receiving antibiotic treatment (91). JHR cannot be predicted reliably, but it was observed that its frequency increases proportionally with white cell count and total protein level in CSF (92). The serofast state refers to a situation in which non-treponemal antibodies decline after treatment but fail to completely revert to a nonreactive state, which may be related to CNS infection (93, 94). Studies have shown that certain genetic factors, such as the strain genotype of 14i/a, host interleukin-10 promoter polymorphisms, differentially expressed cytokines and microRNAs, can predict increased risk of serofast status (94–97). However, regardless of whether it is a risk factor or predictive indicator, there remains uncertainty in the risk or prediction of disease occurrence, as it only represents a possibility with varying degrees of likelihood (98).

## Diagnosis of neurosyphilis

The identification of neurosyphilis typically necessitates the integration of epidemiological information, neurologic or neuropsychiatric manifestations, serologic analysis of blood and CSF, and, in certain instances, imaging assessment. For syphilis patients with neurological symptoms, almost all major guidelines recommend lumbar puncture (99–101). Nonetheless, the neurological symptoms of neurosyphilis are not specific. Early neurosyphilis can affect the meninges and central blood vessels, including syphilitic meningitis, meningovascular neurosyphilis and syphilitic gummas, often manifested as headache, nausea, vomiting, blurred consciousness, and neck stiffness; late neurosyphilis affects the spinal cord and brain parenchyma, including general paresis and tabes dorsalis, manifested as ataxia, impaired memory, disorientation, depression, hallucinations, and mania (102, 103). Furthermore, as a great imitator, neurosyphilis can mimic a wide range of neurological and psychiatric diseases (104), including but not limited to autoimmune encephalitis (105), acute ischemic stroke (106), status epilepticus (107), posterior uveitis (108), asymptomatic optic perineuritis (109). These phenotypes suggest that *T. pallidum* can invade and affect one or more components of the CNS.

Among the diagnostic criteria, CSF examination is necessary. Research on susceptibility to neurosyphilis provides key insights into the pathogenesis and clinical strategies, though many questions still remain, particularly regarding when patients should undergo CSF testing to screen for neurosyphilis. Based on a general survey of relevant guidelines, scholars pay great emphasis to neurological symptoms, syphilis stages, and HIV infection in lumbar puncture (Table 2). As mentioned above, there is a consensus that syphilis patients with neurological symptoms require CSF examination. Additionally, there is limited support for routine lumbar puncture among HIV-positive and HIV-negative persons without neurologic symptoms owing to a lack of evidence that routine lumbar puncture improves clinical outcomes (79). It is necessary to supplement the probability of ANS developing into symptomatic neurosyphilis and the risks and cost-effectiveness of lumbar puncture must be weighed.

**Table 2:** Insights on lumbar puncture in the guidelines of neurosyphilis.

**Guideline**	**Continent/Country**	**Suggestions about lumbar puncture**
Guidelines for diagnosis and treatment of syphilis, gonorrhea, and genital *Chlamydia trachomatis* infection (2020) (110)	China	Lumbar puncture should be performed on all individuals with syphilis and HIV infection to exclude neurosyphilis.
Asian guidelines for syphilis (2022) (99)	Asia	There is no consensus about the need for lumbar puncture in patients with syphilis without any neurological, ocular, or otological symptoms, except for those with tertiary syphilis.
2020 European guideline on the management of syphilis (57)	Europe	CSF assessment is indicated in patients with: -clinical evidence of neurological, ocular, and auricular involvement, whatever the stage of the disease; tertiary syphilis (cardiovascular, gummatous). Some experts still recommend CSF assessment in ANS patients: HIV-positive patients with late syphilis and CD4 cells ≤ 350/mm^3^ and/or a serum VDRL/RPR titer >1:32; in those who have serological failure or are serofast; in those given alternative treatment for late syphilis.
German guidelines on the diagnosis and treatment of neurosyphilis (102)	German	Lumbar puncture is indicated in patients with (at least two out of four are met): CD4 cell count ≤ 200 cells/μl; untreated HIV infection; detectable HIV load; high VDRL titer (>1:64).
UK national guidelines on the management of syphilis 2015 (111)	UK	Routine CSF examination of patients with latent syphilis is not recommended.

ANS, asymptomatic neurosyphilis; CSF, cerebrospinal fluid; HIV, human immunodeficiency virus; RPR, rapid plasma reagin; VDRL, venereal disease research laborator.

A positive CSF venereal disease research laboratory (VDRL) test is considered highly specific for neurosyphilis, whereas a nonreactive CSF fluorescent treponemal antibody absorption (FTA-ABS) test is likely to exclude neurosyphilis (112). The occurrence of false positive results can be attributed to the ability of anti-*T. pallidum* IgG antibodies to traverse the BBB and access the CSF. Consequently, in comparison to non-treponemal tests, the specificity of treponemal tests for CSF examination are lower (57).

Currently, there is no gold standard for diagnosing neurosyphilis, making it difficult to evaluate the diagnostic efficiency of new methods (113). The key to indirect detection lies in distinguishing the specific antibodies synthesized intrathecally, and the antibody index of specific anti-Treponema IgG is a promising new tool (114). PCR lacks sensitivity as a direct detection method (115). At present, there have been no successful development of *T. pallidum* antigen detection kits. The prevailing situations may indicate that *T. pallidum* has the ability to conceal or attach itself to the human body, and the widespread use of antibiotics in other diseases, thus posing a challenge for conventional techniques to detect nucleic acids or antigens in the blood. Overall, application of new technologies with higher sensitivity, such as nested PCR (nPCR) and loop-mediated isothermal amplification (LAMP) assay, may pave the way for the detection of *T. pallidum* in CSF. *T. pallidum* DNA detection rate by LAMP assay was 87.5% in secondary syphilis, which is higher than that in a nPCR study, which achieved *T. pallidum* DNA detection rate respectively in 47.5%, 60.7% of urine sediment and plasma samples from patients with secondary syphilis (116, 117). Actually, researchers have also made many efforts, such as improving sample preprocessing methods and seeking inspiration from case reports to find new sample types (such as saliva) (118, 119).

## Treatment and antibiotic resistance of neurosyphilis

In treatment of neurosyphilis, it is crucial to consider the BBB penetration and effective drug concentrations in CSF (120). The recommended treatment for neurosyphilis is intravenous aqueous crystalline penicillin G (18–24 million units per day, continuous infusion for 10–14 days) (14). Doxycycline and ceftriaxone are considered viable alternatives for penicillin-allergic patients with great BBB penetration ability (121, 122). Doxycycline can be administered orally and can also treat other sexually transmitted infections simultaneously (123). Similar outcomes have been observed in patients with neurosyphilis treated with procaine G penicillin vs. doxycycline (124). Ceftriaxone, by contrast, requires parenteral administration, and its therapeutic effect appears to be controversial (123, 125). A follow-up study suggested that ceftriaxone was associated with a 23% failure rate of treatment for HIV-infected patients with ANS (125). However, another prospective pilot study of HIV-infected patients with ANS has shown no difference in the serologic response to treatment with ceftriaxone vs. procaine penicillin plus probenecid (126). Probenecid can reduce renal tubular secretion and inhibit the active transport of intracranial penicillin by inhibiting Oat3, thereby increasing the bioavailability of penicillin G (127). An enhanced regimen consisting of benzathine penicillin G, ceftriaxone, and doxycycline has demonstrated greater efficacy than the recommended regimen (128). Additionally, a preclinical study has demonstrated linezolid as a promising clinical treatment for syphilis (129). Linezolid is low-cost, safe, and generally well tolerated during short course oral administration, with sufficient concentration in CSF (130). The recent breakthrough in *in vitro* cultivation of *T. pallidum* has facilitated identification of potential candidates for syphilis treatment, as determined by the minimum inhibitory concentration (MIC) (131). Future studies involving neurosyphilis-related strains will provide more convincing evidence for the selection of drugs for the treatment of neurosyphilis.

To date, no clinical manifestations of penicillin resistance have been found in *T. pallidum*, despite reports of increased gene mutations associated with penicillin resistance and that treatment may fail over time (132). Molecular epidemiology study has revealed a growing prevalence of macrolide resistance in *T. pallidum*, which coincides with the increased usage of macrolides due to guideline recommendations (133). In fact, the 2020 European guideline excluded azithromycin as an alternative treatment for syphilis at any stage (57). To tackle the potential crisis of drug resistance, subtractive genomics approaches have been employed to identify salvicine as a potential therapeutic molecule against *T. pallidum* (134).

## Prevention of neurosyphilis

Although penicillin treatment is effective in patients diagnosed with early-stage neurosyphilis, early diagnosis of neurosyphilis is rather difficult, and prevention is particularly important considering the poor prognosis of late-stage patients (135). In addition, given the fatal consequences of congenital syphilis, development of syphilis vaccines is of utmost importance for enhancing public health (136).

The specific significance of the syphilis vaccine for neurosyphilis is to prevent transmission of *T. pallidum* from the infected site and progression to neurosyphilis (137). However, development of a syphilis vaccine is hindered by challenges, including the difficulty of culturing *T. pallidum in vitro*, antigenic mutation in TprK, and the low content and vulnerability of the outer membrane proteins (OMPs); thus, no syphilis vaccine has yet progressed to clinical trials (17, 138). Despite these hurdles, researchers have shown positive responses to this pursuit. To evaluate the efficacy of a syphilis vaccine, a heterologous antigen presentation system (noninfectious *Borrelia burgdorferi*) was designed to express *T. pallidum* antigens (139). Recent advancements in long-term culture systems (by cocultivation with rabbit epithelial cells in a microaerophilic atmosphere) and *in vitro* drug sensitivity testing provide potential solutions for the culture of *T. pallidum* (138). In addition, enrichment techniques that do not rely on culture, or high sensitivity methods, have also been introduced for assays or genetic analyses (140). Vulnerability to outer membrane extraction can be minimized through the use of gel microdroplet techniques, while bioinformatics methods aid in predicting OMPs without the performance of fragile outer membrane (141). Additionally, antigenic variation can be addressed by designing strains that impair the ability to alter TprK (41). The latest field of molecular biology and bioinformatics provides unprecedented opportunities for the identification of new vaccine targets. Several adhesins, such as TP0136 (142), TP0751 (143), and TP0954 (144), have been identified as major vaccine candidates, highlighting their role in invasion and dissemination of *T. pallidum*. Current limitations of these candidates include the requirement of extremely high doses, lack of cross-protection and potential side effects of adjuvants (142, 144). Although some scientists have attempted multicomponent vaccines, complete protection against infection has not yet been achieved (137). Undeniably, inducing partial protection vaccines contributes to the attenuation of transmission and, hence, blockade of progression to neurosyphilis (145). The general topic of vaccines for *T. pallidum* has been thoroughly addressed elsewhere and here will not go into more detail (136, 146).

*T. pallidum* is primarily transmitted through skin-to-skin or mucosal contact during sexual encounters, as well as through vertical transmission (147). Over the past three decades, public attention to sexually transmitted infections has focused primarily on HIV infection, while other infections such as syphilis have gradually been marginalized (148). Preexposure prophylaxis (PrEP) has been, extensively studied as a crucial intervention to prevent HIV transmission but has not been applied to syphilis (149). Interestingly, the incidence of syphilis is higher among MSM taking HIV PrEP than among those who do not in Australia (150). This causal relationship may be attributed to a phenomenon called “risk compensation,” whereby a reduction in HIV risk may cause lax thinking and increased risky behavior (151). However, a German study believes that HIV PrEP was associated with no impact on the prevalence of syphilis among MSM and concerns about risk compensation should not be the barrier to PrEP use in men with behavioral risk for HIV acquisition (152). It is necessary to carry out more studies on the impact of HIV PrEP on the prevalence of syphilis in other countries or regions. These findings underscore the significance of employing combination prevention strategies or developing a syphilis-specific PrEP.

Treatment as prevention (TasP) is an additional method that is effective in controlling the source of infection (153). For HIV-positive patients, it refers to taking HIV medications to prevent sexual transmission of HIV. The proportion of infants born to syphilis mothers suffering from neurosyphilis is higher than expected. For syphilis patients, antenatal treatment has demonstrated high efficacy in reducing the risk of congenital syphilis with the dose of at least 2.4 MU penicillin given at least 28 days before delivery (154). However, a case-control study revealed that treatment of neurosyphilis remains difficult, even if most pregnant women with syphilis are treated with penicillin, and that this is related to inadequate treatment of sexual partners (155). Effective management and control of neurosyphilis continues to face significant obstacles.

## Discussion

Taken together, neurosyphilis denotes an infection of the CNS with a poor prognosis among individuals with syphilis. Although neurosyphilis has been described for centuries, numerous aspects remain unknown (as outlined in Table 3). To address the dearth of epidemiological investigations on neurosyphilis, it is imperative to conduct additional observational studies and consider the implementation of comprehensive surveillance systems on a national or regional scale. As discussed, recent literature increasingly indicates the interrelation between neurosyphilis, the immune response, and genetic factors. However, time is needed for the specific implementation of these strategies in clinical practice. In addition to focusing on the *T. pallidum* itself, pathogen-host interactions should also be taken into account, especially the pathogens with high neuroinvasion properties and host with high susceptibility to infection.

**Table 3:** Important unanswered questions in neurosyphilis.

**Area**	**Questions**
Epidemiology	•How high is the authentic incidence and mortality of neurosyphilis?
		•What is the probability of the ANS patients progressing to symptomatic neurosyphilis?
Pathogenesis	•What is the mechanism of *T. pallidum* invading the CNS? • What is the mechanism of the CNS damage mediated by neurosyphilis?
		•Will HIV infection affect the susceptibility to neurosyphilis?
		•What genetic factors make humans susceptible to neurosyphilis?
Diagnosis	•When do syphilis patients need lumbar puncture to evaluate neurosyphilis?
		•How are specific antibodies produced in CSF of patients with neurosyphilis? How to accurately detect?
		•How to early identify neurosyphilis?
Administration	•What tests can be used to monitor the treatment response of patients with neurosyphilis?
		•Can standard penicillin treatment improve the long-term prognosis of neurosyphilis?
		•Will the increase in gene mutations related to penicillin resistance gradually accumulate, leading to the emergence of penicillin-resistant *T. pallidum*?
		•What are the indications for stopping treatment for neurosyphilis?
		•How to develop a vaccine against neurosyphilis?

ANS, asymptomatic neurosyphilis; CNS, central nervous system; CSF, cerebrospinal fluid.

Currently, clinical suspicion of neurosyphilis primarily arises in patients with syphilis who exhibit neurological and/or psychiatric symptoms. In such cases, it is appropriate to consider serum non-treponemal test titer and serofast status. Accelerating the development of potential serological predictors, standardizing their use, and promptly introducing them into clinical practice would greatly assist clinicians in determining the optimal timing for lumbar puncture. Furthermore, further clinical studies with higher levels of evidence are necessary to explore the efficacy and long-term prognosis of antibiotic therapy and prior to implementation, both the BBB penetration ability and the potential neurotoxicity must be meticulously evaluated.

Given the sustained sensitivity of *T. pallidum* to penicillin, the prevalence of neurosyphilis signifies a disregard for prevention and management, thus necessitating development of vaccines. However, this endeavor faces significant challenges and lags behind the development of vaccines for other bacteria. The latest field of molecular biology and bioinformatics provides unprecedented opportunities for the identification of new vaccine targets. In order to effectively control neurosyphilis, it is also crucial to address the stigma associated with the disease. This involves not only providing proper care for the mental health of patients but also engaging in scientific education. Moving forward, emphasis needs to be placed on the most cost-effective strategies of prevention to mitigate the global burden of neurosyphilis.

## Author contributions

SW: Conceptualization, Visualization, Writing – original draft. FY: Writing – review & editing. YW: Writing – review & editing. DL: Supervision, Writing – review & editing.
